# Real-world assessment of longitudinal opioid use and healthcare resource utilization in patients undergoing colorectal resection

**DOI:** 10.1016/j.sopen.2024.05.022

**Published:** 2024-05-31

**Authors:** Gary Schwartz, Jennifer H. Lin, Swapnabir Kakoty

**Affiliations:** a Maimonides Medical Center, 4802 Tenth Avenue, Brooklyn, NY, United States of America; b AABP Integrative Pain Care, LLC, Brooklyn, NY, United States of America; c Pacira BioSciences, Inc., 5401 W Kennedy Blvd, Suite 890, Tampa, FL, United States of America

**Keywords:** Colorectal surgery, Opioid utilization, Length of stay, Persistent postdischarge opioid use, Inpatient readmission

## Abstract

Liposomal bupivacaine (LB) has been used in multimodal pain management regimens to improve postsurgical analgesia. This retrospective cohort analysis assessed clinical and economic outcomes of LB vs non-LB analgesia in minimally invasive colorectal resection surgery using real-world patient data from the IQVIA linkage claims databases. Patients who received LB were 1:1 matched to patients who did not receive LB (non-LB) via propensity scores. Outcomes included opioid use during the perioperative (2 weeks before surgery to 2 weeks after discharge), continued (>2 weeks to 3 months after discharge), and persistent (>3 months to 6 months after discharge) periods and healthcare resource utilization (HRU) during the first 3 months after discharge. Mean opioid consumption was lower in the LB (*n* = 4397) versus non-LB (n = 4397) cohort perioperatively (483 vs 538 morphine milligram equivalents [MMEs]; *P* = 0.001) and after discharge within ∼3 months (222 vs 328 MMEs; *P* < 0.0001) and 3–6 months (245 vs 384 MMEs; *P* < 0.0001). The LB cohort had shorter mean length of stay (5.2 vs 5.7 days; *P* < 0.0001) and fewer inpatient readmissions (odds ratio [OR], 0.71; *P* < 0.0001), emergency department visits (OR, 0.78; *P* < 0.0001), and outpatient/office visits (OR, 0.91; *P* = 0.028) than the non-LB cohort 3 months after discharge. These data suggest use of LB in minimally invasive colorectal resection surgery may reduce perioperative and postdischarge opioid use as well as HRU. Although additional studies are needed to confirm these findings, this analysis provides valuable real-world data from large claims databases to evaluate clinical and economic outcomes that complement other types of retrospective and prospective studies.

Colorectal resection (CR) is a common surgical procedure performed in the United States with patients reporting moderate-to-severe pain in the postoperative period; notably, gastrointestinal surgery has the third highest prevalence of chronic postsurgical opioid use [[1], [2], [3]]. Clinical practice guidelines for colorectal surgery recommend use of multimodal pain protocols to limit opioid consumption [4]. Liposomal bupivacaine (LB) is a long-acting bupivacaine formulation that can provide prolonged analgesia and reduce opioid consumption via local infiltration, interscalene brachial plexus nerve blocks, sciatic nerve blocks in the popliteal fossa, or adductor canal blocks [5]. Previous studies suggest that multimodal pain management regimens for CR, including use of LB, may provide benefits regarding postsurgical analgesia and healthcare resource utilization (HRU) [6,7]. However, data are limited regarding opioid use after hospital discharge in patients undergoing CR. We used a retrospective claims data analysis approach to assess long-term, real-world opioid use and HRU among patients undergoing CR who did or did not receive LB for postsurgical analgesia.

The deidentified IQVIA linkage claims databases include inpatient and outpatient data with patient-level demographic, procedure, and diagnosis records as well as pharmacy prescription and medical claims data [8]. Data were analyzed from adult patients who underwent inpatient minimally invasive primary CR (January 1, 2016, to June 30, 2019). Outcomes included opioid use in morphine milligram equivalents (MMEs) during the hospital stay and after discharge. Opioid use was measured for multiple time periods: (1) total perioperative period (2 weeks before surgery to 2 weeks after discharge), including 72 h after surgery and the total inpatient stay; (2) continued period (>2 weeks to 3 months after discharge); and (3) persistent period (>3 months to 6 months after discharge). Postdischarge all-cause HRU outcomes over 90 days included inpatient readmission rates, emergency department visit rates, and outpatient/office visits assessed at 1, 2, and 3 months after discharge. Each patient receiving LB was matched to 1 patient receiving non-LB analgesia with a propensity score obtained by regressing the treatment (ie, LB) probability against 9 observed characteristics [9].

The LB and non-LB cohorts (4397 patients each) were balanced across all characteristics after propensity score matching with a standardized difference of <10 % for all measured variables. Overall, both cohorts had a mean age of 61 years and more female than male patients. Approximately 30 % of patients in both cohorts were exposed to opioids before surgery. Most hospitals were in urban areas (>98 %), with approximately two-thirds of hospitals located in the South.

Patients who received LB consumed significantly fewer opioids than the non-LB cohort during the total perioperative period (mean difference, −54 MMEs [95 % confidence interval (CI), −86 to −22 MMEs]; *P* = 0.001), including significant reductions in total opioid consumption during the 72-h postsurgical period (mean difference, −21 MMEs [95 % CI, −41 to −1 MMEs]; *P* = 0.035) and the total inpatient period (mean difference, −34 MMEs [95 % CI, −62 to −7 MMEs]; *P* = 0.013) (Fig. 1). Significant reductions in opioid use were also seen after discharge, including throughout the continued (>2 weeks to 3 months after discharge; mean difference, −106 MMEs [95 % CI, −157 to −55 MMEs]; *P* < 0.0001) and the persistent (>3 to 6 months after discharge; mean difference, −138 MMEs [95 % CI, −200 to −77 MMEs]; *P* < 0.0001) periods. In subgroup analyses, the rates of opioid consumption at different time periods in the opioid-naive and opioid-experienced groups were similar to the overall results. There was no interaction of cancer history on the associations between LB treatment and opioid consumption during multiple time periods (Wald test *P* for interaction ≥0.11 across time periods).

**Fig. 1:**
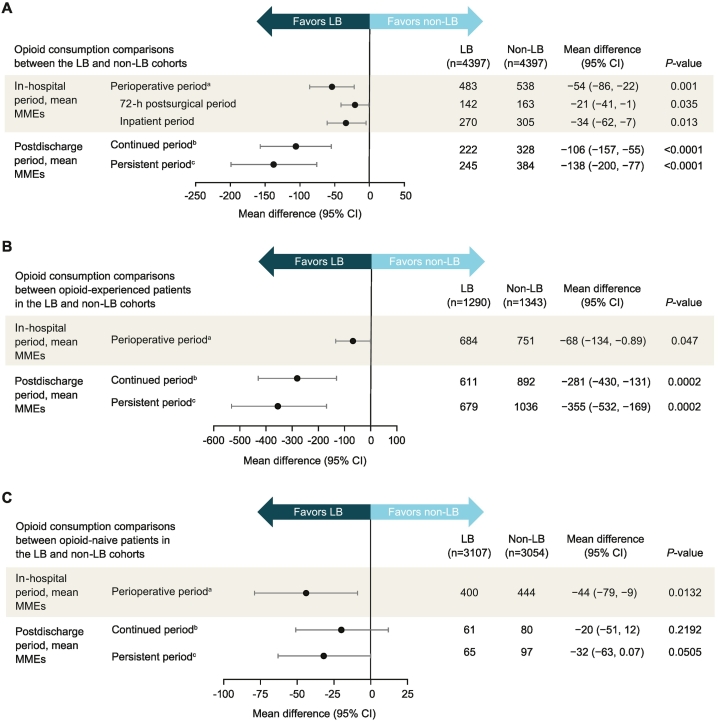
(A) Opioid consumption comparisons between the LB and non-LB cohorts. (B and C) Opioid consumption comparisons between the LB and non-LB cohorts according to prior opioid status. CI, confidence interval; LB, liposomal bupivacaine; MME, milligram morphine equivalent. ^a^2 weeks before surgery to 2 weeks after discharge. ^b^>2 weeks to 3 months after discharge. ^c^>3 to 6 months after discharge.

The LB cohort also had an ∼0.5-day shorter hospital stay (5.2 vs 5.7 days; *P* < 0.0001; rate ratio, 0.93 [95 % CI, 0.91–0.95]; *P* < 0.0001). At hospital discharge, the LB cohort had 35 % lower odds of being transferred to a care facility (3.4 % vs 5.2 %; odds ratio, 0.65 [95 % CI, 0.53–0.81]; *P* < 0.0001). During the postdischarge 1-, 2-, and 3-month time points, the LB cohort had 29 % to 34 % lower odds of inpatient readmissions, 15 % to 22 % lower odds of emergency department visits, and 9 % to 17 % lower odds of outpatient/office visits relative to the non-LB cohort (*P* ≤ 0.028 across all endpoints and time points; Fig. 2).

**Fig. 2:**
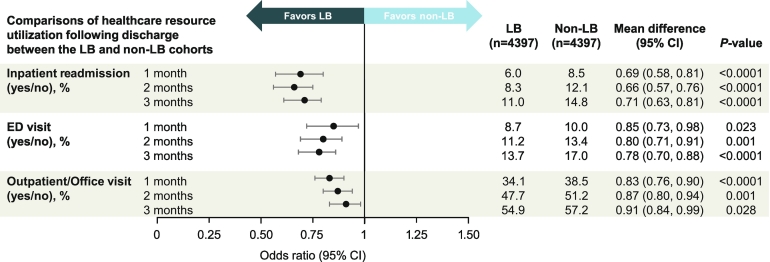
Comparison of healthcare resource utilization following discharge between the LB and non-LB cohorts. CI, confidence interval; ED, emergency department; LB, liposomal bupivacaine.

Claims-based analyses such as the current study offer several strengths. These data sets can encompass a large sample from a wide range of hospitals across the United States; for example, the IQVIA linkage claims databases comprise patient-level information from >300 healthcare facilities, providing a large sample representing real-world outcomes. Claims databases with prescription information and longitudinal follow-up in patients also provide valuable information on chronic opioid use in patients exposed to opioids during surgery who may be vulnerable to opioid misuse. Our findings about opioid use up to 6 months after hospital discharge are consistent with prior research indicating that patients undergoing major surgeries such as CR are susceptible to developing long-term opioid use [3,10]. Additionally, long-term HRU data provide insight into economic burden after discharge, including but not limited to postsurgical pain and complications. However, secondary analyses of large administrative databases are limited by several factors. For example, availability of clinical information is limited or incomplete (eg, elective vs nonelective surgery, intraoperative and postsurgical pain regimens, administered anesthetics and dosing). It is also unclear whether filled prescriptions have been consumed by patients. Moreover, the databases are subject to potential data entry errors and misclassifications. No firm causality can be established on the basis of the observed associations, while the risk of residual confounding caused by extraneous variables remains.

This retrospective analysis demonstrates the value of claims database analyses for understanding real-world opioid use and HRU after CR. Our results suggest the use of LB for CR may help reduce postsurgical opioid requirements resulting in decreased future opioid consumption, better pain management, and reduced HRU, which is likely to translate into lower healthcare costs. Overall, there is a need for long-term follow-up of patients undergoing CR to determine the impact of LB on opioid consumption and overall HRU. The present findings warrant replication in other studies.

## Funding sources

This analysis was supported by Pacira BioSciences, Inc. Writing and editorial assistance was funded by Pacira BioSciences, Inc. The study sponsor was involved in the collection, analysis, and interpretation of the data, the writing of the manuscript, and the decision to submit this article for publication.

## Ethics approval

This analysis was exempt from institutional review board review requirements per US Department of Health and Human Services policy (Title 45 Code of Federal Regulations, Part 46 of the United States) because patient records in the IQVIA linkage claims database are deidentified.

## CRediT authorship contribution statement

**Gary Schwartz:** Writing – review & editing, Conceptualization. **Jennifer H. Lin:** Writing – review & editing, Methodology, Formal analysis, Conceptualization. **Swapnabir Kakoty:** Writing – review & editing, Methodology, Formal analysis, Conceptualization.

## Declaration of competing interest

GS has received consultancy fees from Pacira BioSciences, Inc., and Dorsal Health. JHL and SK are employees of Pacira BioSciences, Inc., and may hold stock or stock options in the company.

## Data Availability

Data for this study were available to the authors via third-party license from IQVIA, a commercial data provider in the United States, and Pacira, which has a license for analysis of the de-identified linkage data. As such, the authors cannot provide the raw data; however, other researchers may access the data by purchase through IQVIA. The data set outputs from the analysis of raw data are also available to qualified researchers upon reasonable request by contacting Jennifer Lin (Jennifer.Lin@pacira.com).
